# Uncovering the unique characteristics of the mandible to improve clinical approaches to mandibular regeneration

**DOI:** 10.3389/fphys.2023.1152301

**Published:** 2023-03-17

**Authors:** Ana Prates Soares, Heilwig Fischer, Sabrin Aydin, Claudius Steffen, Katharina Schmidt-Bleek, Carsten Rendenbach

**Affiliations:** ^1^ Department of Oral and Maxillofacial Surgery, Charité—Universitätsmedizin Berlin, Corporate Member of Freie Universität Berlin, and Humboldt-Universität zu Berlin, and Berlin Institute of Health, Berlin, Germany; ^2^ Julius Wolff Institute for Biomechanics and Musculoskeletal Regeneration, Berlin Institute of Health at Charité—Universitätsmedizin Berlin, Berlin, Germany; ^3^ Centrum für Muskuloskeletale Chirurgie, Charité—Universitätsmedizin Berlin, Corporate Member of Freie Universität Berlin, and Humboldt-Universität zu Berlin, and Berlin Institute of Health, Berlin, Germany; ^4^ BIH Biomedical Innovation Academy, BIH Charité Clinician Scientist Program, Berlin Institute of Health at Charité—Universitätsmedizin Berlin, Berlin, Germany; ^5^ Berlin Institute of Health Centre for Regenerative Therapies (BCRT), Berlin Institute of Health at Charité—Universitätsmedizin Berlin, Berlin, Germany

**Keywords:** mandible, morphophysiology, free tissue flap, mandibular reconstruction, morphogenesis, bone regeneration

## Abstract

The mandible (lower jaw) bone is aesthetically responsible for shaping the lower face, physiologically in charge of the masticatory movements, and phonetically accountable for the articulation of different phonemes. Thus, pathologies that result in great damage to the mandible severely impact the lives of patients. Mandibular reconstruction techniques are mainly based on the use of flaps, most notably free vascularized fibula flaps. However, the mandible is a craniofacial bone with unique characteristics. Its morphogenesis, morphology, physiology, biomechanics, genetic profile, and osteoimmune environment are different from any other non-craniofacial bone. This fact is especially important to consider during mandibular reconstruction, as all these differences result in unique clinical traits of the mandible that can impact the results of jaw reconstructions. Furthermore, overall changes in the mandible and the flap post-reconstruction may be dissimilar, and the replacement process of the bone graft tissue during healing can take years, which in some cases can result in postsurgical complications. Therefore, the present review highlights the uniqueness of the jaw and how this factor can influence the outcome of its reconstruction while using an exemplary clinical case of pseudoarthrosis in a free vascularized fibula flap.

## 1 Introduction

Various pathologies can affect mandibular health, such as congenital deformities, cysts, tumors, infections, necrosis, as well as trauma (Slootweg, 2010; Pickrell et al., 2017). In large tumors or osteonecrosis of the jaws, a significant portion of the mandible may need to be surgically resected. The resulting discontinuity of the mandible alters its morphology and function, which severely impacts the lives of patients physiologically, aesthetically, and psychologically. The intricate geometry, sizeable dimensions, and complex mechanics of the mandible complicate the search for bone engineering alternatives for mandibular reconstruction. Another difficulty in this endeavor is the fact that most bone replacement materials and most reconstructive techniques are designed for and tested in appendicular bones (Fernandez de Grado et al., 2018).

Current useful regenerative treatments for segmental mandibular resections rely on autologous bone flaps from the iliac crest, scapula, and most favorably the fibula. Due to the fibula’s limited weight-bearing function in the leg, its ample length, and location, allowing for a two-team surgical procedure (simultaneous resection and flap harvesting), the free vascularized fibula flap (FFF) has become the main surgical approach for mandibular reconstruction (Antúnez-Conde et al., 2021). The clinical use of the FFF technique has allowed for reasonably positive results in dental and prosthetic rehabilitation, speech intelligibility, deglutition function, the aesthetic outcome, and the subjective overall wellbeing of tumor patients post-rehabilitation (Attia et al., 2019). However, even the use of FFF results in several severe complications in 12.4%–20% of cases (Verhelst et al., 2019; Knitschke et al., 2021). Flap postoperative issues can range from early- and late-onset plate-related abscess formation, fistula, plate exposure, and delayed bone healing, to non-union. Consequently, the patient can lose proper mandibular function.

The mandible is unlike any other bone used for its reconstruction. There are differences between the mandible and other bones (appendicular and most flat bones) in morphology, biomechanics, and physiology, as well as morphogenesis, cell genotype and phenotype, the osteoimmune environment, and several clinically relevant aspects. Therefore, the present work aims to investigate the possible influence of the characteristics that distinguish the mandible from other bones on clinical reconstructive work and the development of new regenerative alternatives, while using a pseudoarthrosis case to illustrate the problem.

## 2 Clinical case example of osseous non-union after FFF reconstruction

**FIGURE 1:**
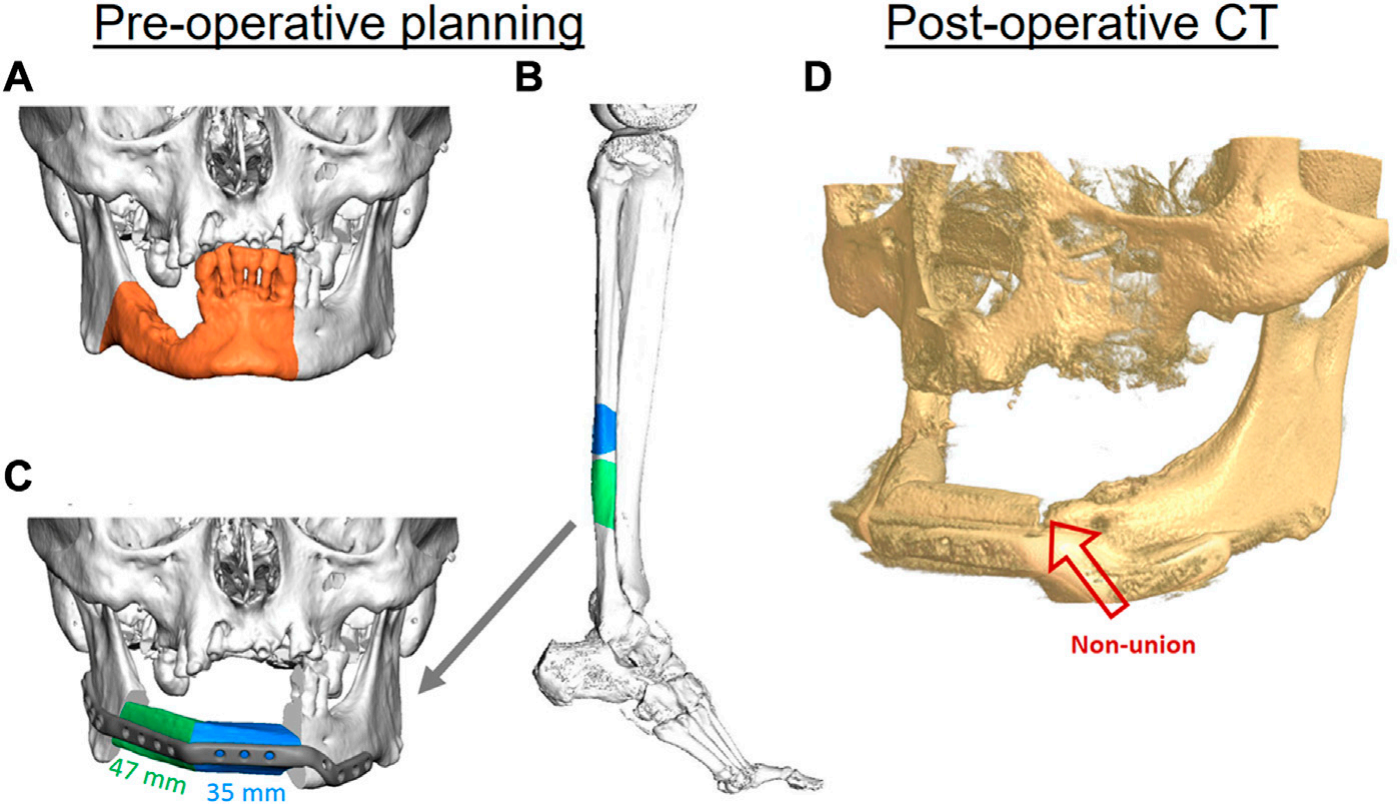
The virtual planning steps for mandibular reconstruction. Three-dimensional (3D) reconstruction of a cone beam computed tomography (CBC) scan, showing the area of affected bone including a safety margin of the right-sided mandible (orange area) **(A)**. A two-segmental fibula free flap (FFF) from the right leg (green and blue parts) was virtually planned for the reconstruction of the mandibular defect using computed tomography (CT) **(B)**. The segments were virtually fitted, and fixation was planned using a patient-specific 3D-printed titanium reconstruction plate **(C)**. The postoperative CBCT scan of the patient after 11 months demonstrates a non-union between the FFF and the mandible in the anterior region (red arrow) **(D)**.

As an example of a typical pseudarthrosis (non-union) case of FFF, a partial union between the fibula and mandible can occur. In this case, a 70-year-old male patient was initially diagnosed and later treated at the department of Oral Maxillofacial Surgery, Charité—Universitätsmedizin Berlin, for oral squamous cell carcinoma without associated metastases. Secondary diagnoses were hypertension, type 2 diabetes, carotid artery stenosis, and alcohol and nicotine abuse. Consequently, tumor resection including segmental mandibular resection, neck dissection, tracheotomy, and mandibular reconstruction were virtually planned. A patient-specific titanium plate (Karl Leibinger Medizintechnik GmbH & Co. KG, Mühlheim an der Donau, Germany) and a microvascular free fibula flap (FFF) with two segments were virtually designed using the computer-aided design and manufacturing (CAD/CAM) workflow to ensure surgical precision and suitable reconstruction (Figure 1).

Postoperatively, the patient received adjuvant radiotherapy performed over a period of 6 weeks with the application of 56 Gy. Postoperative controls using computed tomography (CT) and clinical examination did not reveal any signs of recurrence within the first 2 years after radiotherapy. Osseous union at the posterior (fibula *vs*. mandible) and intermediate gaps (fibula *vs*. fibula) showed satisfactory results; However, there were still signs of incomplete osseous union in the anterior gap connecting the mandible with the FFF after 12 months (Figure 1D). The removal of fixation plates and screws is generally necessary before dental implant placement for oral rehabilitation. In this case, oral rehabilitation with dental implants was not possible within the first 2 years after surgery and radiotherapy due to the incomplete osseous union. To try to solve the incomplete union, bone grafting from the iliac crest was performed 3.5 years after the initial reconstruction. However, the incomplete union persisted, the plate had to be kept in place, and oral rehabilitation with dental implants still could not be performed. The result was a limited number of teeth in the patient’s mouth, which impaired his masticatory and speech capacities.

In the presented case, many factors may have contributed to the flap complication that resulted in an impaired functional outcome, such as age, radiotherapy, and co-morbidities. Despite the patient’s overall health issues and habits, the distal area of the fibula did bridge with the mandible. While there was sufficient healing between the two fibula segments in the anterior area, there was diminished bone healing between the fibula and mandible, even though both intersegmental gaps were located correspondingly in the mandible and thus underwent similar biomechanics. This begs the question as to whether the differences in healing are caused by biological differences between the mandible and fibula bone.

## 3 Uncovering the differences

### 3.1 Morphogenesis

The most noticeable difference between craniofacial bones, like the mandible, and hard tissues in the rest of the body is their embryonic origin. While the mandible stems from the cranial neural crest, bones from the limbs have a mesodermal origin (Yuan and Chai, 2019). In contrast to mesodermal cells, cranial neural crest cells originate from a broader variety of tissues (Chai and Maxson, 2006) with ectodermal and mesenchymal natures (Zalc et al., 2021), such as smooth muscles, teeth, sensory neurons, and craniofacial bones.

The developmental mechanism of the mandible also differs from other skeletal bones. Appendicular bones, for example, are solely formed through endochondral ossification, a multistage process that first generates a cartilage template, that is, then converted into bone. On the other hand, the lower jaw is formed as a mosaic (Hinton et al., 2017). The most posterior (condyle and angle) and the most anterior (symphysis) areas of the mandible are formed through endochondral ossification, while the bulk of the mandible is formed by intramembranous ossification, a process in which an osteoid tissue is deposited, then mineralized and does not require a cartilage template (Hinton et al., 2017; Yuan and Chai, 2019).

### 3.2 Morphophysiology and biomechanics

**FIGURE 2:**
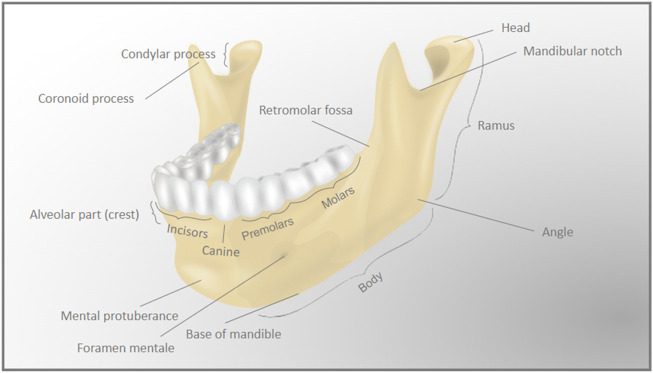
Detailed anatomy of the mandible. The complex anatomy of the mandible (lower jaw) bone is represented by the differently labeled anatomic regions.

Like other craniofacial bones, the mandible has a complex shape. The mandible is arch-shaped in its anterior portion, called the body, and has two vertical extensions on its posterior ends, the rami. The lower part of the body is the base of the mandible, while the superior part of the body is the alveolar process, which holds the lower teeth. The anterior area of the body forms a triangle, called the mental protuberance, and it is where the fusion of the two lateral halves of the mandible occurs in early infancy (Nyström and Ranta, 2003). The bilateral most posterior parts of the body form the angles of the mandible with the rami. The rami extend vertically upwards, and each ramus forms a condyle at its most posterior ends. Each condyle (left and right) articulates with its respective temporal bone to form the temporomandibular joints (Figure 2).

It is through the articulation of the temporomandibular joint that the body of the mandible works as a cantilever, while both joints move in an intricate and coordinated three-dimensional (3D) manner. Normal masticatory and speech functions result in frequent loading of the mandible with variable amplitude and force (Grigoriadis et al., 2014). The complexity of the mandibular movements results in the deformation of its structure. Most impressively, during the opening, protrusion (frontal excursion), and lateralization (lateral excursion) movements, the mandible arch becomes narrower, only returning to its original form during centric mouth closing movements (El-Sheikh et al., 2007). Therefore, the shape and constant 3D deformation of the mandible make its biomechanical environment different from that of appendicular bones.

Likewise, there are daily higher, more rhythmic, and predictable compressive strains in the lower jaw bone than in appendicular bones (de Jong et al., 2010). Because bone regeneration is sensitive to loading cycles and the strain rate (Rubin and Lanyon, 1984), the differences between the mandible and the original mechanical environment of the flaps may play a role in bone healing after mandibular reconstructive surgeries. Furthermore, the masticatory muscle fibers have a different profile from those of the trunk and limb muscles. Directly related to the constant motion of the mandible either during function (e.g., chewing, speaking, and swallowing) or parafunction (e.g., grinding, clenching, and thumb suction), masticatory muscles have higher fatigue resistance and increased muscle force (Sciote et al., 2003). This indicates an added difference in the mechanical load at the mandibular site, which will demand changes in the macro- and microstructure of the flaps, as there is a direct link between morphology and biomechanics in bone (Glatt et al., 2017).

For its biomechanical demands, the mandibular structure must be more flexible to withstand daily load-bending movements. The unique constant multidirectional movements of the mandible lead to differences between this and other axial and appendicular bones. The collagen content of the mandible has been shown to be higher and has less post-translational modification (Lys hydroxylation) than that of humeral and femoral bones (Sasaki et al., 2010), which makes the lower jaw bone more flexible. The higher collagen content and lower hydroxylation are also connected to a higher bone toughness, or ability to deform without fracture. Furthermore, bone mineral density (BMD), which looks at the mean mass of a bone in a certain area to assess the fragility or strength (quality) of a bone, seems to be higher in the lower jaw bone body and symphysis areas than in the femur, hip, or spine (Drage et al., 2007).

The microstructure of the mandible also differs from that of other bones. Compared with the iliac, the cortical bone of the mandible has a smaller vessel surface to bone volume ratio, and therefore a lower vessel porosity (Rothweiler et al., 2022). Bone mineralization density distribution (BMDD) is used to measure bone mass distribution on the microscale. BMDD indicates areas of higher and lower mineral apposition. Moreover, the lacunar-canalicular network (LCN) is the group of small cavities (lacuna) in the bone that house osteocytes and their minute dendritic extensions that form interconnecting channels (canaliculi), through which neighboring cells can communicate. The BMDD and lacuna distribution have a more heterogeneous spatial distribution in the mandible than in the tibia or femur (Hesse et al., 2014). There is a higher bone density surrounding the mandibular LCN (Hesse et al., 2015). Furthermore, the total lacunar volume is larger in mandibular samples (Hesse et al., 2014), and there is a higher canalicular volume and canalicular length, and more nodes in the younger mandibular LCN (Bortel et al., 2022). However, the impact of this greater cellular communication in the mandible compared with possible flaps is still uncertain.

### 3.3 Direct comparison between the mandible and the fibula

**FIGURE 3:**
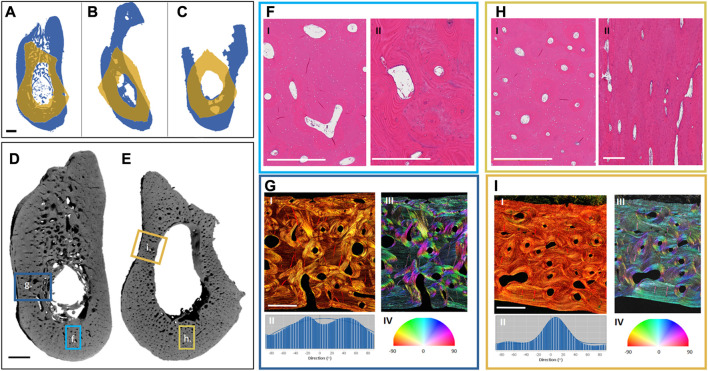
Preliminary analysis of differences between the mandible and the fibula. Projections of the exemplary cross-sections of three different patients **(A–C)**: The mandibular projections in blue and the overlaying projections of the corresponding fibulas in yellow, showing the differences in the shape and size of the bones. Three different fibula shapes can be noted: Irregular **(A)**, triangular **(B)**, and quadrilateral **(C)**. Renderings of one of the patient samples reveal the different complexity in the morphology of the mandible **(D)** and the fibula **(E)**. Highlighted hematoxylin and eosin **(H, E)**-stained regions from the mandible **(F)** and fibula **(H)**—Cross-sections of the mandible **(F, I)** and fibula **(H, I)**—Reveal a more homogeneous size and distribution of the Haversian canals in the fibula, and a larger vascular area in the mandible. Further, regional longitudinal sections in the mandible **(F, II)** and fibula **(H, II)** again show a more regular longitudinally aligned Haversian system in the fibula. Highlighted Picrosirius red-stained regions from the mandible **(G, I)** and fibula **(I, I)** were analyzed for the directionality of their fiber bundles **(G, II, I, II)**, showing higher multi-directionality in the mandible **(G, II, G, III, G, IV)** than in the fibula **(I, II, I, III, I, IV)**. The black scale bar is 2 mm and the white scale bar is 500 µm.

The free vascularized fibula flap (FFF) is the main surgical approach for mandibular reconstruction; it is chosen due to its ample length and mostly triangular cross-section, similarly to the mandible (Ide et al., 2015). However, geometrically, the fibula presents major disadvantages for mandibular rehabilitation, as its lower height can hinder implant-prosthetic rehabilitation, and its linear shape cannot mimic the round angles of the lower jaw (Antúnez-Conde et al., 2021) (Figures 3A–C).

To better understand the underlying differences in the bones which we aim to join together by using the FFF technique, a preliminary look at the morphological patterns of mandible and fibula bones was undertaken. Exemplary mandibular and fibular cross-sections were harvested from three different patients (under Charité Universitätsmedizin Ethical committee approval EA1/062/21) undergoing mandibular reconstruction using FFF. The samples were harvested from the edges of the resection during surgery. They were then imaged using a laboratory micro-CT (Skyscan 1172, Bruker, pixel size: 9 µm) and histologically processed and stained with hematoxylin and eosin as well as Picrosirius red.

The macroscopic examination of the samples revealed that the fibular sections have a thicker cortical area, no trabecular bone, and corresponded to between 1/2 and 2/3 of the mandibular height (Figures 3D, E). Each fibula sample could be distinctively classified based on its shape as irregular (Figure 3A), triangular (Figure 3B), or quadrilateral (Figure 3C) (Ide et al., 2015). Microscopically, there is a similar osteocyte density (number of osteocytes per bone area) in the mandibular and fibular samples and a higher vascularity (vessel area per bone area) in the cortex of the mandible. Furthermore, the Haversian system distribution is different between samples (Figures 3F, H). There is greater homogeneity in the fibula with a dominance of longitudinally aligned Haversian canals (Figure 3F). In contrast to the fibula, the Haversian system in the mandible presents a more irregular pattern in both orientation and shape (Figure 3H). Furthermore, the organization of the collagen fiber bundles revealed by Picrosirius red staining is different between the bones (Figures 3G, I). While the fibula possesses unidirectional wide layers of collagen fibers on the outer and inner circumference of the cortical bone (Figures 3I,I–IV), the mandible shows a more irregular collagen pattern (Figures 3G,I–IV). These morphological differences are most likely connected to the biomechanical environment of each sample. Because this is a preliminary comparison of the morphology of both bones, a more ample assessment is needed with multiple samples and their surgical follow-up to assess the impact of different morphologies on the clinical results. Further description of the methodology used for the morphological assessment can be found in the Supplementary Material.

### 3.4 Gene expression

**FIGURE 4:**
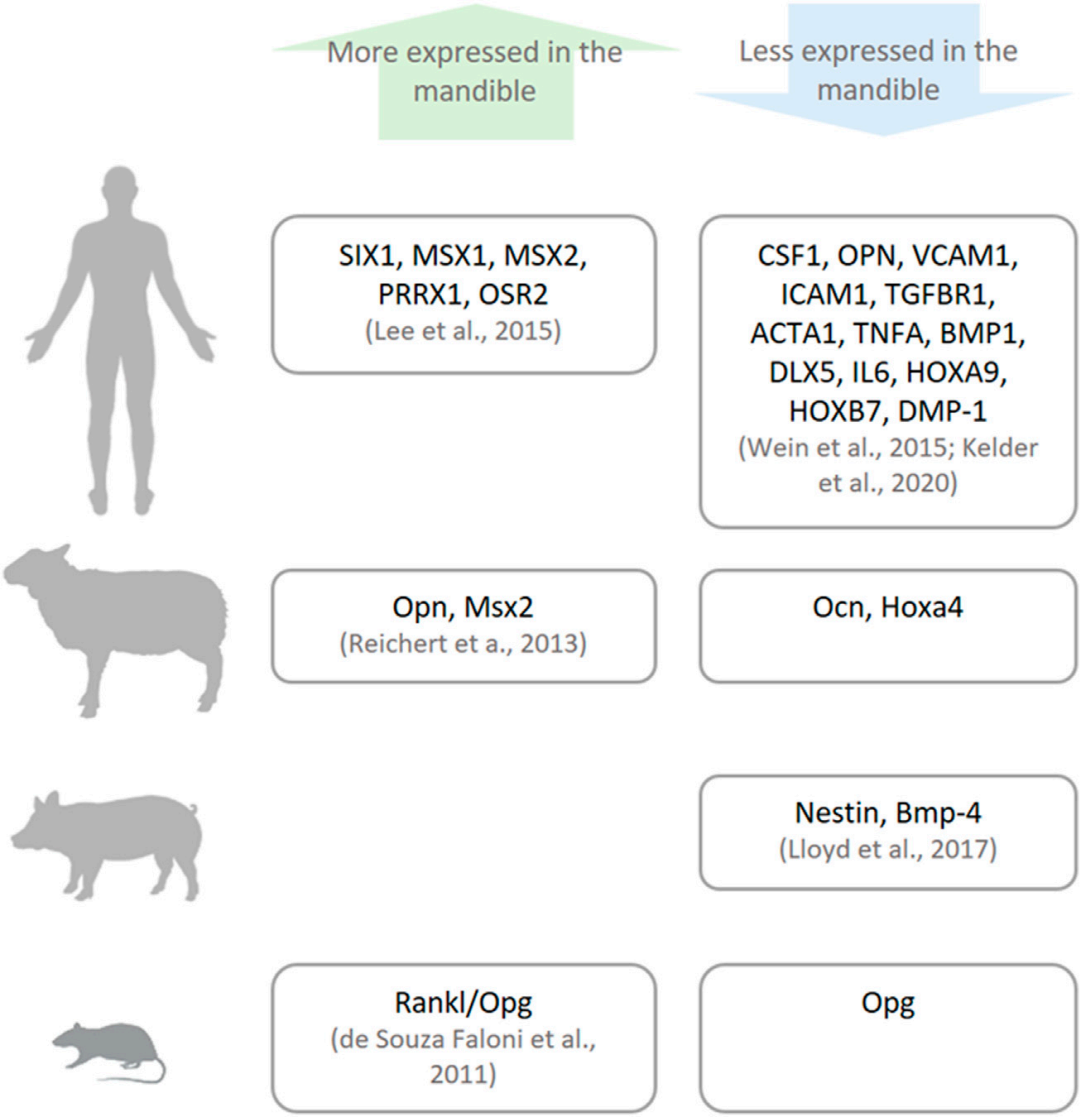
Genetic differences between the mandible and other bones. There are differences in the expressions of specific genes (named in the boxes) in humans, sheep, pigs, and small animals (rats and mice) as reported in the literature.

The gene expression profile differs between bone sites (Isaac et al., 2017; Youlten et al., 2021) and at times between species (Figure 4), most notably, the expression of genes that function as morphogenesis regulators and cell differentiation, called homeobox genes (Mark et al., 1997). Homeobox genes are divided into two subfamilies: clustered (Hox) genes, which provide cells with regional information along the body axis and are highly expressed in appendicular bones, and non-clustered (Hox negative) genes (e.g., MSX, PAX, and DLX), which are present in different tissues and possess multiple functions including craniofacial morphology (Leucht et al., 2008). Hox stem cells harvested from appendicular bones and grafted into a mandibular defect are able to keep their Hox profile (Leucht et al., 2008). This discrepancy between the Hox status of the mandible and grafted cells lingers throughout the bone healing process, and as result, the transplanted cells differentiate into chondrocytes instead of osteoblasts, locally ensuring an atypical endochondral ossification instead of an intramembranous one (Leucht et al., 2008). On the other hand, when Hox-negative cells from the mandible are transplanted into tibial defects, they can change their Hox profile (Leucht et al., 2008). This higher plasticity of mandibular cells can be a result of their embryonic origin that stems from broader multipotent cranial neural crest cells.

Besides the difference in morphogenic genes, the mandible and appendicular bones in the human body can also diverge in their genetic profile. Lee et al. (2015) confirmed higher expression of craniofacial morphogenesis–related genes (non-clustered Homeobox genes: SIX1, MSX1, MSX2, PRRX1, and OSR2) in the mandible compared with the iliac bone. Moreover, the iliac bone and tibia show higher expression of genes related to the proliferation and differentiation of stem cells into the osteolineage (Hox genes: HOXB7 and HOXA9); osteoblast differentiation (DLX5 and DMP-1; Wein et al., 2015); cartilage formation and maintenance (BMP1 and TGFBR1; Wein et al., 2015); osteoclast differentiation, activation, and their attachment to the extracellular matrix (CSF1, ICAM1, VCAM1, OPN; Wein et al., 2015; Kelder et al., 2020); and structural cytoskeletal component of osteocytes (ACTA1; Kelder et al., 2020). Taken together, the difference in gene expression signals the more mature and differentiated character of cells from appendicular bones. The dissimilarity in gene profiles between bone cells may influence their divergent response to stimuli.

A well-researched clinical example of the difference between the response of the mandible and appendicular bones to stimuli is the reaction to chronic consumption of bisphosphonates, a medication used to prevent bone loss. In the mandible, bisphosphonates induce the suppression of the MSX1 and OPN genes and a subsequent increase in DLX5 expression (Wehrhan et al., 2011). MSX1 suppression limits the proliferation of pre-osteoblasts (Roybal et al., 2010; Wehrhan et al., 2011) and also causes an increase in the expression of DLX5, which in turn induces osteoblast terminal differentiation and further extracellular matrix mineralization (Zhang et al., 2003; Wehrhan et al., 2015). OPN depletion impairs osteoclast attachment to the bone matrix (Wehrhan et al., 2015) as well as the migration of endothelial cells and, therefore, the early development of blood vessels (Wein et al., 2019). Moreover, although both the mandible and appendicular bones express OPN, only one type of RNA splice variant is expressed in the mandible (OPNa), while two others are expressed in appendicular bones (OPNb and OPNc) (Wein et al., 2019). This difference might be one of the reasons that bisphosphonates specifically impact the blood perfusion of the mandible. Together, MSX1 and OPN suppression generates a highly mineralized extracellular matrix, with a lack of efficient bone turnover and low blood perfusion, therefore resulting in tissue death characteristically present in the pathomorphology of bisphosphonate-related osteonecrosis of the jaws (BRONJ) (Koerdt et al., 2014). Perhaps due to the lack of deleterious impact on appendicular bones, FFF can be used in mandibular reconstruction of osteonecrosis cases with a >90% success rate (Sacco et al., 2018).

In vertebrates used as animal models in bone research, gene expression also varies between the mandible and appendicular bones, although not necessarily in the same pattern as their human orthologs. Specifically, the mandible from murine, porcine, and ovine origins have been harvested and their gene expression evaluated and compared with their appendicular bone counterparts. In rodents, the Rankl/Opg ratio, which positively regulates osteoclast activation, is higher in mandibular marrow-derived cells (de Souza Faloni et al., 2011). In porcine bone marrow cells, there is higher expression of genes related to bone development (Nestin) and limb formation (Bmp-4) in appendicular bones (Lloyd et al., 2017). On the other hand, in ovine bones, genes related to craniofacial morphogenesis (Msx2) and osteoclast attachment to the extracellular matrix (Opn) are more expressed, while genes related to bone remodeling (Ocn) and limb morphogenesis (Hoxa4) are less expressed in the lower jaw (Reichert et al., 2013). The differences between species and their gene expression may be caused by evolutionary epigenetic regulation (Chan et al., 2018).

### 3.5 *In vitro* dynamics and *in vivo* applications

**FIGURE 5:**
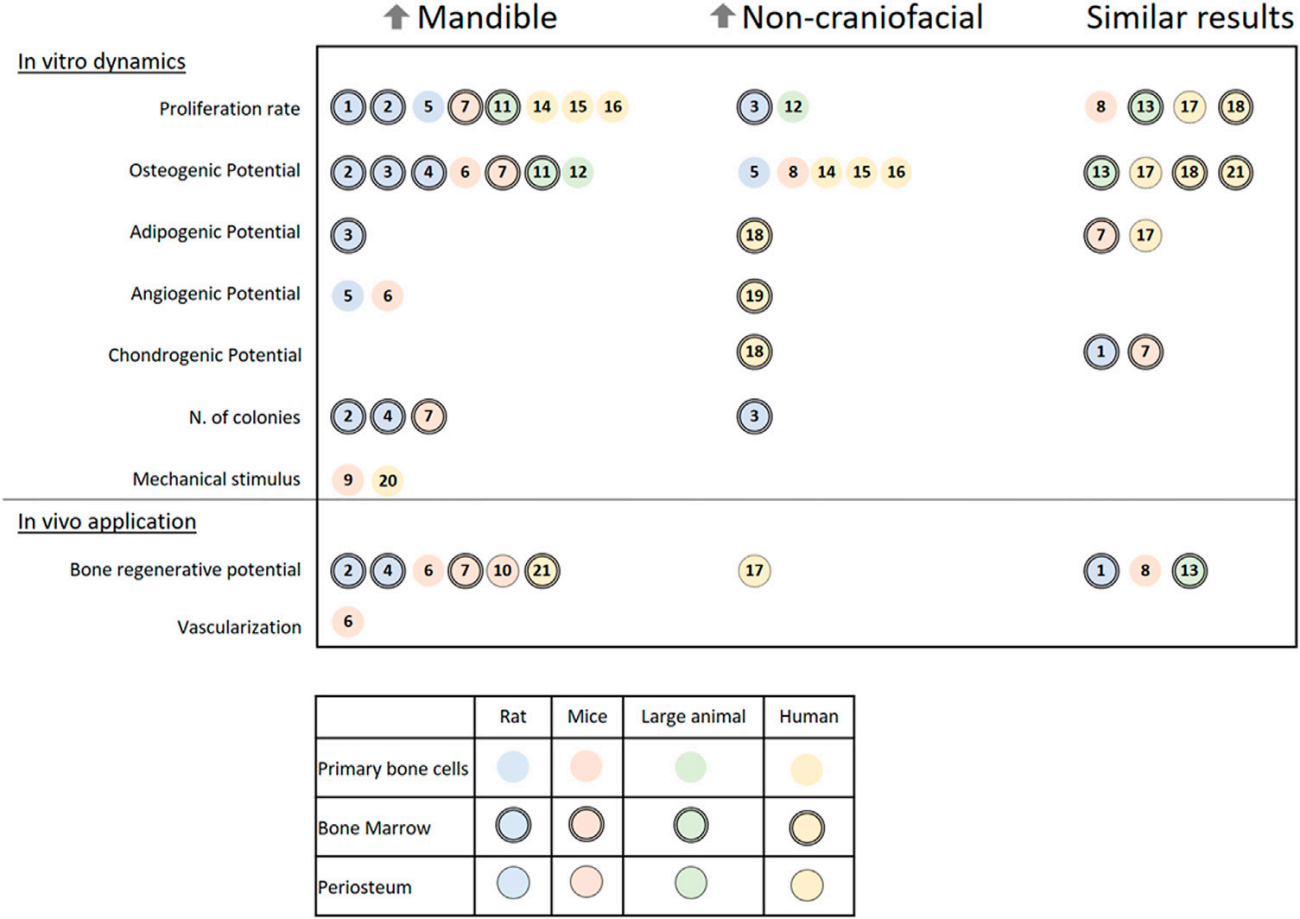
A literature review on the *in vitro* dynamics and *in vivo* applications of different cells harvested from the mandible and non-craniofacial bones. The results are separated into three columns referencing papers (a total of 21 papers) in which cells from the mandible of rats, mice, large animals, or humans presented higher activity (↑Mandible) or lower activity (↑Non-craniofacial) or similar activity (Similar results) when harvested from the same individual and compared in the same conditions to non-craniofacial bones cells. Symbols indicate the origin of the cells: Rats (blue), mice (rosa), large animal models (green), humans (yellow), primary bone cells (open circle), bone marrow cells (double contoured circle), and periosteum (single contoured circle). Numbered references: 1) 10.1177/2041731419830427, 2) 10.1007/s00441-014-1927-4, 3) 10.1089/scd.2019.0256, 4) 10.1177/0022034510378427, 5) 10.1007/s10735-019-09810-6, 6) 10.1016/j.jbo.2020.100346, 7) 10.1177/0022034510387796, 8) 10.1002/jbm4.10382, 9) 10.1016/j.yexcr.2011.07.015, 10) 10.1111/jre.12229 11) 10.1016/j.archoralbio.2017.01.012, 12) 10.1016/j.gene.2013.04.026, 13) 10.1177/0022034518772283, 14) 10.1016/j.jcms.2015.07.030, 15) 10.1007/s00784-014-1353-8, 16) 10.3390/ijms21145072, 17) 10.3389/fcell.2020.554984, 18) 10.1359/JBMR.041117, 19) 10.1016/j.acthis.2015.02.006, 20) 10.1016/j.jobcr.2019.09.005, 21) 10.1111/j.1601-0825.2007.01402.x.

Numerous studies have tested and compared the characteristics of cells harvested from the mandible of various species to corresponding cells from appendicular or flat bones (non-craniofacial bones). As shown in Figure 5, the outcome from *in vitro* and *in vivo* assessments of skeletal cells can vary, which further demonstrates the need to standardize methods to examine possible reasons for the distinct results. For better understanding in this review, the results from different papers have been classified by cell origins as primary bone cells, periosteum cells, or bone marrow cells, and by the studied species divided into rats, mice, large animal models (comprising sheep, dogs, and pigs), or humans.

The first characteristic that most researchers examine is the proliferation rate of the isolated cells. In the majority of papers, cells harvested from the mandible present a higher proliferation rate than of any other bone (Yamaza et al., 2011; Lee et al., 2015, Lee et al.,2019; Wein et al., 2015; Lloyd et al., 2017; Yang et al., 2019; Kelder et al., 2020). Important exceptions are connected to the difference in medium formulation (Li et al., 2020) and the comparison between cells from juvenile bone (Reichert et al., 2013). A colony-forming unit assay has also been used in a few studies and has shown that mandibular cells have a higher cloning ability (Aghaloo et al., 2010; Yamaza et al., 2011; Dong et al., 2014).

When assessed, the osteogenic potential of cells harvested from the mandible varies among the studied species. The majority of small (rats and mice) and large animal models have shown a higher osteogenic potential in cells harvested from the mandible (Aghaloo et al., 2010; Yamaza et al., 2011; Reichert et al., 2013; Dong et al., 2014; Lloyd et al., 2017; Li et al., 2020; Eber et al., 2021). Meanwhile, in humans, cells harvested from appendicular and flat bones mostly have a higher osteogenic potential (Wein et al., 2015; Kelder et al., 2020), although it is sometimes similar to that of cells harvested from the mandible (Groeneveldt et al., 2020). Only one report showed higher osteogenic potential in human mandibular bone marrow stem cells (BMSCs; Stefanik et al., 2008); Note that compared with other studies, these authors used a higher concentration of fetal bovine serum (FBS; 20%) in the growth medium. Not much can be said about the comparison of the adipogenic, chondrogenic, or angiogenic potentials of the cells, as only some of the studies have presented data about it (Matsubara et al., 2004; Yamaza et al., 2011; Marini et al., 2015; Lee et al., 2019; Groeneveldt et al., 2020; Li et al., 2020; Eber et al., 2021) with confounding results. Furthermore, when mechanically stimulated by orbital shear stress, there is an increase in osteogenesis, pro-angiogenic cytokines, and mineralization of cells harvested from the mandible *in vitro* (Pravitharangul et al., 2019). When stimulated by ultrasound, the same type of cells from mice increase their receptor activator of nuclear factor kappa-Β ligand (RANKL) expression, a gene related to the increase of osteoclastic activity (Watabe et al., 2011). The results of both mechanical *in vitro* studies indicate that local remodeling of the mandible is mechanically modulated, maybe even more so than in other bones.

The expression of distinct genes and proteins can vary at different time points between the mandible and non-craniofacial bone tissue *in vitro*. Their expression dynamics reveal that the cells diverge in response to osteogenic induction. Importantly, there is a greater increase in alkaline phosphatase (ALP) activity in cells harvested from the mandible of small and large animals (Yamaza et al., 2011; Reichert et al., 2013; Dong et al., 2014; Lloyd et al., 2017). This enzyme is one of the most used markers for osteogenic differentiation. Moreover, osteocalcin (OCN), which encodes an essential protein for apatite crystallites alignment and bone strength (Moriishi et al., 2020), has higher expression in *in vitro* cells harvested from the rat mandible (Aghaloo et al., 2010; Dong et al., 2014; Lee et al., 2019; Li et al., 2020).

All the differences between cells harvested from the mandible and non-craniofacial bones *in vitro* can impact their application in a tissue engineering context. The characteristics that distinguish the mandible might even require site-specific cell choice (Reichert et al., 2013). Researchers have used small animal models to test the bone regenerative potential of the isolated cells. They have mixed the cells with various materials (e.g., gelatin and β-tricalcium phosphate) and then applied the mixtures to the animals either subcutaneously (Aghaloo et al., 2010; Yamaza et al., 2011; Groeneveldt et al., 2020; Son et al., 2020; Eber et al., 2021) or into bone defects (Stefanik et al., 2008; Dong et al., 2014; Ichikawa et al., 2015; Wang et al., 2018; Lee et al., 2019). The results revealed that cells harvested from the mandible produce more mineralized tissue content than cells harvested from non-craniofacial bone (Aghaloo et al., 2010; Yamaza et al., 2011; Dong et al., 2014; Ichikawa et al., 2015; Eber et al., 2021).

### 3.6 Osteoimmunology

Interactions between immune and bone systems, termed the osteoimmune environment, play an essential role in regulating bone health. Both innate and adaptive immune cells are associated with the dynamic processes of bone turnover and can be classified through their cluster of differentiation (CD), meaning the expression of different surface molecules (Actor, 2019). Most remarkably, macrophages, which are highly plastic innate immune cells, give rise to essential bone cells: osteoclasts. Macrophages are also present in all bone healing phases, having either a more pro-inflammatory profile (so-called M1 macrophages) and expressing CD86^+^ on its surface, or a more bone-regenerating profile (also called M2 macrophages) with CD206^+^ surface expression (Schlundt et al., 2021). T cells with different CD play a dynamic role in bone homeostasis (Pacifici, 2016). While activated CD4^+^ CD8^+^ T cells and CD4^+^ T helper 17 (Th17) can increase bone resorption, regulatory CD4^+^ T cells (Tregs) can inhibit bone resorption. Activated B cells are also implicated in increased bone resorption (Horowitz et al., 2010).

**FIGURE 6:**
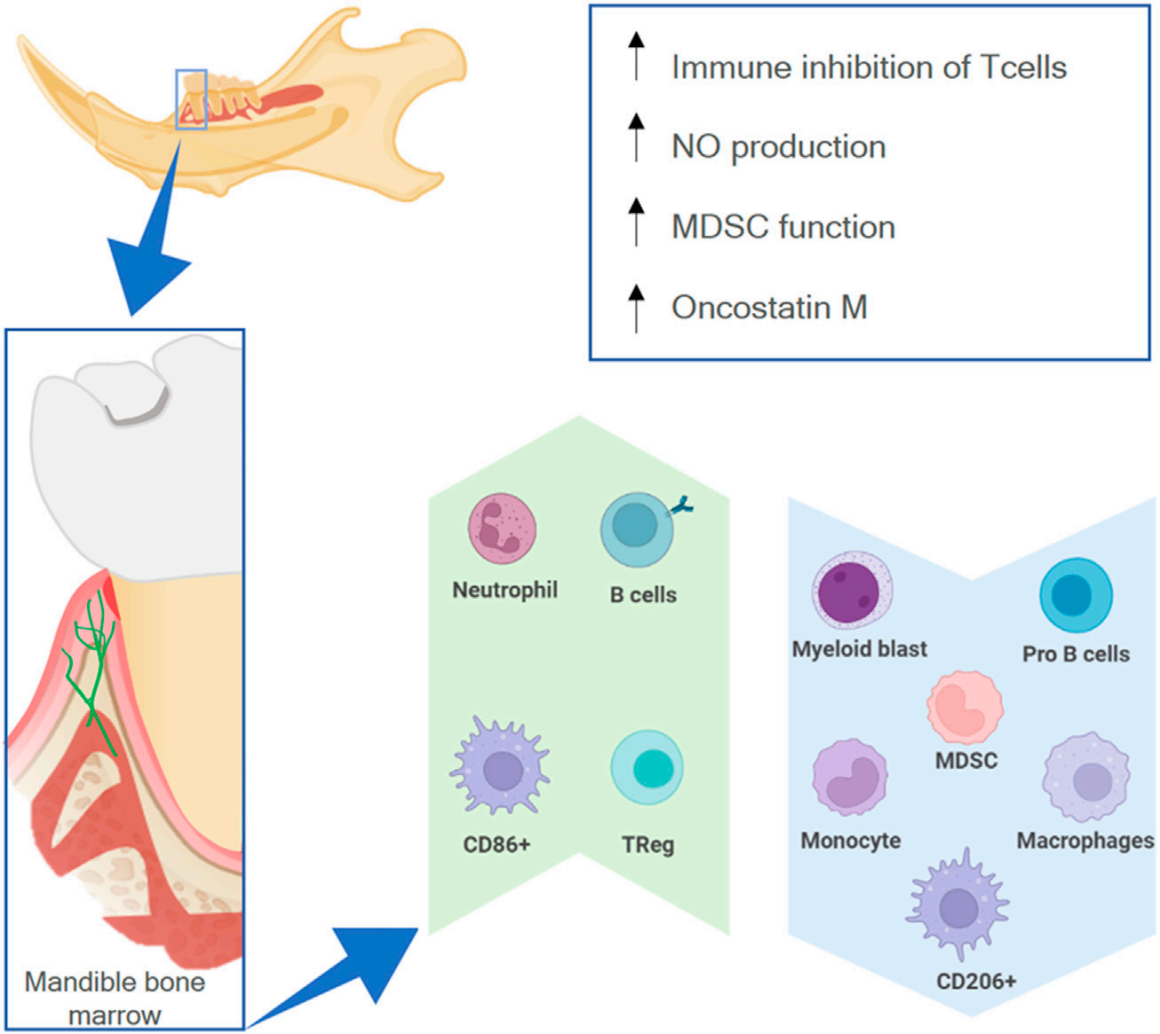
The osteoimmune environment in the mandible. The high permeability of the oral mucosa allows external antigens to have contact with the mandibular marrow (lower box), resulting in a higher expression (green upwards arrow) or lower expression (blue downwards arrow) of different immune cells, as well as higher activity of specific processes (upper box).

The mandible has a different osteoimmune microenvironment from that of non-craniofacial bones (Figure 6). Anatomically, the existence of a permeable barrier between the oral cavity and the periodontal ligament (called junctional epithelial attachment) as well as the presence of lymphatic vessels circulating from the oral mucosa—Colonized by a wide range of microbes—To the bone marrow allow microbial products to constantly reach the mandibular marrow space (Jensen and Folke, 1974; Barker, 1982). Therefore, the bone marrow of the mandible is constantly exposed to antigens from the oral microbiota and has a more active immune environment than non-craniofacial bones, which only have contact with bacterial products during infections or when transported by the vasculature (Hathaway-Schrader et al., 2022).

Due to the environment in the oral cavity, there are differences between the myeloid cell population in the mandible and in non-oral bones. There are fewer monocyte progenitor cells like myeloid lineage-early blasts and myeloid blasts as well as fewer monocytes, myeloid-derived suppressor cells (MDSCs), and macrophages (Cd11b + F4/80+) (de Souza Faloni et al., 2011; Kwack et al., 2021) in the mandibular marrow. Furthermore, BMSCs from the mandible of mice show high nitric oxide production (Yamaza et al., 2011). There is a higher inhibitory capacity of CD4^+^ CD8^+^ T cells, more Tregs, an increase in MDSC function, and more B cells (Kwack et al., 2021). These features demonstrate a higher immunosuppressive potential and more adaptive humoral response in the mandibular environment. This might be necessary because commensal microbiota can provoke a higher pro-inflammatory response in alveolar bone marrow cells than on appendicular bone marrow cells, increasing the amount of activated dendritic cells, CD4^+^ T cells, and Th17 cells (Hathaway-Schrader et al., 2022). Moreover, there are more CD86^+^ and fewer CD206+ macrophages in the mandible, which also signals a more pro-inflammatory response to microbial products (Lin et al., 2021).

Macrophages/monocytes can also elicit a greater modulatory effect on mesenchymal stem cells (MSCs) in the mandibular context. Macrophage conditioned medium has a greater effect on the proliferation, colony formation, and osteogenic potential of MSCs harvested from the mandible (Lin et al., 2021). Furthermore, macrophages harvested from the mandible have a higher *in vitro* capacity for recruiting MSCs (Dong et al., 2014) as well as a higher expression of oncostatin M (Osm) (Lin et al., 2021). Osm is a cytokine that can regulate the osteogenic fate of MSCs (Guihard et al., 2015) and stimulate osteoblasts to secrete RANKL, which in turn results in more active osteoclasts in the mandible (Lin et al., 2021).

The osteoclastic population of the mandible also differs from that of non-craniofacial bones. Morphologically, osteoclasts from the mandible are larger (de Souza Faloni et al., 2011), longer, and have a smaller amount of nuclei (Azari et al., 2011; de Souza Faloni et al., 2011; Goldberg, 2016). *In vitro*, when compared with appendicular bones, the osteoclastogenesis from mouse mandibular marrow cells is slower (Goldberg, 2016), while cells from the bone marrow of human mandibles have a lower osteoclastogenic potential (Kelder et al., 2020). For rats and mice, there is a similar resorptive activity of mandibular and non-craniofacial bone osteoclasts (de Souza Faloni et al., 2011; Goldberg, 2016); however, osteoclasts from human mandibles have a lower resorptive potential (Kelder et al., 2020). The substrate can modulate osteoclastic activity, and when mandibular osteoclasts are seeded in dentin they grow larger in size and number (de Souza Faloni et al., 2011). Furthermore, osteoclasts from the mandible can respond differently to hormonal changes. In culture under the influence of parathyroid hormone (PTH), osteoclastogenesis of mandibular bone marrow cells is lower than that of non-craniofacial bones (Chaichanasakul et al., 2014), and *in vivo*, the loss of ovarian hormones does not appear to affect the osteoclastic content (Goldberg, 2016).

The assembled data on osteoclasts seem to underline the difference between bones as well as between osteoclastic activity in humans and other species. As reviewed by Sculean et al. (2019), mandibular bone remodeling after tooth extraction in large animal models (e.g., dogs and monkeys) is faster than in humans. Additional data from the literature also reveal a lower remodeling potential of human mandibles at the cellular level compared with appendicular bones (Kelder et al., 2020). Moreover, although it is customary to state that bone turnover of the mandible is higher than of appendicular bones due to the results of animal studies (Huja et al., 2006; Inoue et al., 2019), this does not seem to be applicable to a broad human population, as there is high variability between individuals (Trombelli et al., 2008). As shown in different clinical studies, bone turnover of the mandible can be similar to that of different appendicular bones (Ristow et al., 2014a; Ristow et al., 2014b).

### 3.7 Clinical aspects

Due to the mandible’s role in the physiology of multiple body functions and the morphology of the lower third of the face, rehabilitating this bone is of the utmost importance. To achieve good surgical results, understanding the susceptibility of the mandible to different ailments and how its healing process works is essential. Furthermore, knowing the differences and similarities between the mandible and the bones used for its regeneration is important for a better long-term outcome of mandibular reconstruction.

Several researchers have investigated, and some have compared, appendicular bone and mandibular healing using small animal models. Overall, these papers have shown similarities between the mandible and appendicular bones regarding their healing processes. Bone fractures in both the mandible and appendicular bones respond to unstable fixation by secondary healing and cartilage formation (Wong et al., 2021; Ito et al., 2022), while stable fixation leads to primary healing and greater intramembranous ossification (DeConde et al., 2014; Wong et al., 2021). To mimic the same physical conditions of tooth extraction in appendicular bone, drill holes can be made with the removal or preservation of the periosteum (Liu et al., 2020; Ito et al., 2022). In rats as in humans, the extraction socket consistently heals through intramembranous ossification as the remaining periodontal cells go through osteoblastic differentiation (Cardaropoli et al., 2003; Trombelli et al., 2008; Ito et al., 2022); However, the ossification speed of the socket is highly variable and individual (Trombelli et al., 2008). Furthermore, drill holes seem to heal faster in the rat tibia than in the rat mandible (Liu et al., 2020). Although drill holes in appendicular bones with and without periosteum mainly heal through intramembranous ossification, there is associated cartilaginous formation in the wound when the periosteum is present, which demonstrates a higher chondrogenic potential in long bones (Ito et al., 2022).

Different techniques can be used for mandibular bone augmentation. Distraction osteogenesis is a surgical technique used to induce changes in bone morphology (height or thickness) by gradual separation and controlled bone formation within the interval space of surgically severed bones. Although used in the mandible and appendicular bones with a similar protocol, distraction osteogenesis has a higher complication rate in appendicular bones (Shah et al., 2021). The reason might be that the distraction osteogenesis process stimulates the focal adhesion kinase (FAK) signaling pathway that is naturally active in cranial neural crest cells during development, and this reversion to a more primary state of the cells can in turn facilitate regeneration in the mandible (Ransom et al., 2018). Another bone augmentation technique is bone grafting. It can be used for horizontal (width) or vertical (thickness) mandibular augmentation. Bone grafts can be harvested from the mandible itself (angle or symphysis) or most commonly from the iliac bone. When mandibular grafts are used, there is a slower vertical loss of the graft; furthermore, they have a lower incidence of peri-implantitis (Kang et al., 2015) and a higher implant survival rate (Sbordone et al., 2009). However, when grafts larger than 5 mm are needed, iliac bone grafts can lead to better results, as they have a structure that allows greater blood perfusion, a necessary trait for larger grafts (Troeltzsch et al., 2016; Rothweiler et al., 2022).

Many changes occur in the organism throughout the aging process that affect bone morphophysiology. Although aging greatly impacts appendicular and flat bones, it does not have the same effect on the mandible. For example, protein-energy malnutrition is a common condition in elderly people; It is mainly caused by appetite loss and ingestion difficulties connected to different age-related ailments (Price, 2008). Protein undernutrition can have an impact on human bone health, leading to bone mass loss and an increase in fractures (Rizzoli and Bonjour, 2004). Appendicular bones are more affected by undernutrition than the mandible in both adult and newborn rats (Nakamoto and Miller, 1977; Nakamoto and Miller, 1979; Mavropoulos et al., 2007). To our knowledge, there is no report on the undernutrition effect on the mandibular structure in humans (Algra et al., 2021).

Another age-related ailment is osteoporosis. The impact of osteoporosis on the mandibular structure of patients is not yet understood. There is no clear evidence that estrogen deficiency induces osteoporotic changes in human mandibles (Nicolielo et al., 2017). Meanwhile, small animal ovariectomized models have shown that in the same individuals, the femur and the tibia are more affected, and thus have a greater decrease in the bone volume fraction and BMD as a consequence of ovariectomy (Mavropoulos et al., 2007; Hsu et al., 2016). Adipogenesis in bone marrow cavities and the formation of bone marrow adipose tissue (BMAT) are also associated with aging and osteoporosis. There is a markedly higher BMAT increase in appendicular bones of ovariectomized rats than in the mandible (Coutel et al., 2019). Furthermore, titanium screws implanted in the mandibles of ovariectomized rats present higher osseointegration than screws implanted in their femurs (Liu et al., 2020). Independent of gender, in humans affected by osteoporosis, there is a moderate correlation between changes in the BMD of reference bones (femur, forearm, and lumbar bones) and the mandible (Horner et al., 1996; Esfahanizadeh et al., 2013). However, the impact of osteoporosis in the mandible is less than in appendicular and flat bones (Horner et al., 1996; Mavropoulos et al., 2007; Esfahanizadeh et al., 2013; Hsu et al., 2016; Nicolielo et al., 2017; Liu et al., 2020). Tooth loss more than aging impacts the morphology of the mandible (Oettlé et al., 2016), without any alterations to the mandible’s BMD (Springe et al., 2014).

The most documented adverse effect that specifically affects oral bones is medication-related osteonecrosis of the jaws (MRONJ). Although MRONJ is the topic of diverse literature reviews, its pathophysiology is not yet fully understood. It is considered a rare occurrence, but many clinical reports have demonstrate the development of MRONJ in patients using antiresorptive medications and angiogenic inhibitors. The most prevalent triggering factors to the development of MRONJ are tooth extraction and spontaneous onset of osteonecrosis (Fliefel et al., 2015). There are a few *in vitro* and *in vivo* reports directly comparing the effects of such medication on the mandible and appendicular bones. *In vivo* reports have shown that the use of zoledronic acid, a potent bisphosphonate applied in numerous diseases to decrease bone resorption, has a targeted deleterious effect in rat and mouse mandibles. Serial injections of zoledronic acid result in three deleterious effects: 1) A decrease in the number of mandibular marrow cells (Wang et al., 2019); 2) Lower expression of mandibular bone turnover signaling promoters (RANKL/OPG and Wnt-3); and 3) Overall suppression of alveolar bone remodeling (Gong et al., 2017), more so in the mandible than in the maxilla (Wang et al., 2019). In contrast, zoledronic acid enhances bone quality during bone remodeling in appendicular bones (Gong et al., 2017). *In vitro* tests using human cells taken from the mandible, iliac, and other appendicular bones and placed under the influence of pamidronate, another potent bisphosphonate, have shown that BMSCs more than bone-derived primary cells can be influenced by this medication (Marolt et al., 2012). The influence of pamidronate *in vitro* leads to a decrease in the survival and metabolic activity of mandibular BMSCs as well as an increase in osteoclastic recruitment compared with iliac BMSCs (Stefanik et al., 2008). After implanting these cells into bone defects in mice, the authors found greater bone formation but with a less organized structure when compared with BMSCs from the iliac bone (Stefanik et al., 2008). These results show the difficulty in ascertaining the cause of MRONJ. The human body, the complex interaction between different systems, and the differences at the individual level are hard to mimic in experimental settings. However, as mentioned earlier in this review, there are genetic clues as to why MRONJ develops particularly in oral bones.

Differently from non-oral bones, the mandible can be affected by odontogenic tumors, which originate from the tooth and periodontal tissues. Moreover, the incidence of distinct bone tumors is different in the mandible. The occurrence of osteochondroma and intraosseous lipoma, for example, is lower than in appendicular bones (Xi et al., 2008; Cakarer et al., 2009; Angiero et al., 2011), and osteochondroma presents a better prognosis in the mandible.

All the aforementioned clinical differences can lead to dissimilar responses from the host and harvested bone to the mechanical and biological environment of the mandible. Observing and differentiating the two bone areas can be important for clinical follow-up, to understand and diagnose their changes. It is especially important to have these clinical differences in mind, because creeping substitution, or the replacement process of the bone graft tissue by new locally formed bone tissue, can take years (Roberts and Rosenbaum, 2012), and cells harvested from long bone can keep their genetic profile at the graft-receiving site (Leucht et al., 2008). Although the clinical success rate of FFF is high, changes in both bone areas post-reconstruction may be dissimilar. More research is needed to investigate microstructural changes and how the mandible and the graft respond to aging and comorbidities over time.

## 4 Conclusion

As this review has shown, there is a large assembly of data pointing to the uniqueness of the physiology, pathology, and clinical needs of the mandible. The main differences between the mandible and other bones can be summarized as follows.

1. While the mandible stems from the cranial neural crest, bones from the limbs have a mesodermal origin.
2. The morphophysiology of the mandible and its associated muscles are different from appendicular bones mainly due to daily higher, more rhythmic, and predictable compressive strains and thus unique biomechanics.
3. The gene expression profile differs between bone sites, especially between morphogenic genes.
4. Cells harvested from the mandible of different species can behave in a dissimilar manner when tested *in vivo* and applied *in vivo.*
5. The mandible has a more active osteoimmune microenvironment than that of non-craniofacial bones.
6. Aging, comorbidities, and pathologies affect the mandible in a dissimilar manner compared with other bones.

Although researchers have uncovered differences between the mandible and other bones, there are still essential questions concerning mandibular biology and its response to stimuli. The underlying differences between the fibula and mandible could have an impact on osseous healing in cases of pseudarthrosis after segmental mandibular reconstruction. Additional studies are needed to closely investigate the causes that lead to diminished healing after osseous reconstructions.
