# Supplementary material for: Uncovering the unique characteristics of the mandible to improve clinical approaches to mandibular regeneration
Source: Front Physiol. 2023 Mar 17;14:1152301. doi: 10.3389/fphys.2023.1152301 (PMC10063818; doi:10.3389/fphys.2023.1152301)
Supplement: Supplementary file 2 [file Presentation1.pdf]

Supplementary Material

Uncovering the unique characteristics of the mandible to improve clinical approaches to mandibular regeneration

Ana Prates Soares, Heilwig Fischer, Sabrin Aydin, Claudius Steffen, Katharina Schmidt-Bleek, Carsten Rendenbach

*** Correspondence:** Corresponding Author: ana.prates-soares@charite.de

1 Sample preparation, imaging and analysis:

For the direct preliminary comparison between mandible and fibula bones, patients' samples (n= 03) were harvested during free vascularized fibula flap mandible reconstruction (under Charité Universitätsmedizin Ethical committee approval EA1/062/21). The samples were then fixated using Paraformaldehyde 4% for 4 days. After fixation, the samples were scanned using a laboratory micro-CT (Skyscan1172, Bruker micro CT, Kontich, Belgium) with a 9µm pixel size (data shown in the paper). Thereafter, the samples were demineralized (EDTA), dehydrated in an ascending alcohol series, and embedded in paraffin. After embedding, the samples were sliced with a 5 µm thickness in cross and longitudinal sections and placed in glass slides, the serial slices were later stained with Hematoxylin Eosin or Picrossirius Red.

Micrographic images were made of the slides (Leica microscope DM6B, Leica Microsystems, Germany) creating mosaic pictures with bright field illumination for the H&E stained samples and polarized light for the Picrossirius stained samples. Cross-section images were then analyzed using ImageJ (version 1.53q, National Institute of Health, USA).

The cortical area of the fibula and the mandibular were analyzed for the presence of vases (vases area/cortical bone area) and the number of osteocytes (number of osteocytes/cortical bone area). For the assessment of vases the H&E images (Supplementary Figure 1a) were converted into 8 bits (Supplementary Figure 1b), filtered using a median filter, and thresholded (Supplementary Figure 1c). For the quantification of osteocytes, the 8bits images were thresholded, the vases were mathematically filtered out, and the number of osteocytes was calculated by using Analyze particles module (Supplementary Figure 1d.).

For the analysis of the cortical collagen fibers organization, selected areas of the Picrossirius red stained images from each sample were cropped and aligned with the medulla on the bottom and the surface on the top (Supplementary Figure 2a). The images were converted into 8 bits (Supplementary Figure 2b) and analyzed using OrientationJ, which generated the color map with the different directions of the fibers (Supplementary Figure 2c). Furthermore, Analyze Directionality was also used in the cropped images to generate the histogram with the different directions of the fibers (between 90° and -90°) (Supplementary Figure 2d).

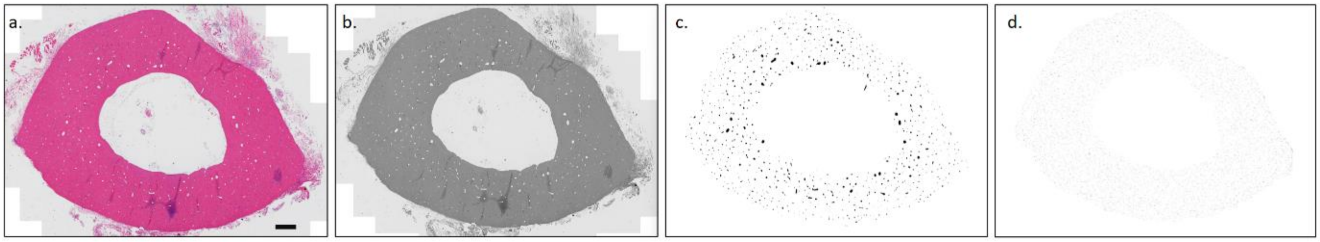

Supplementary Figure 1. Image processing steps for the assessment of the vase area and the osteocyte amount on the cortical bone of a fibula sample. Scale bar: 1mm.

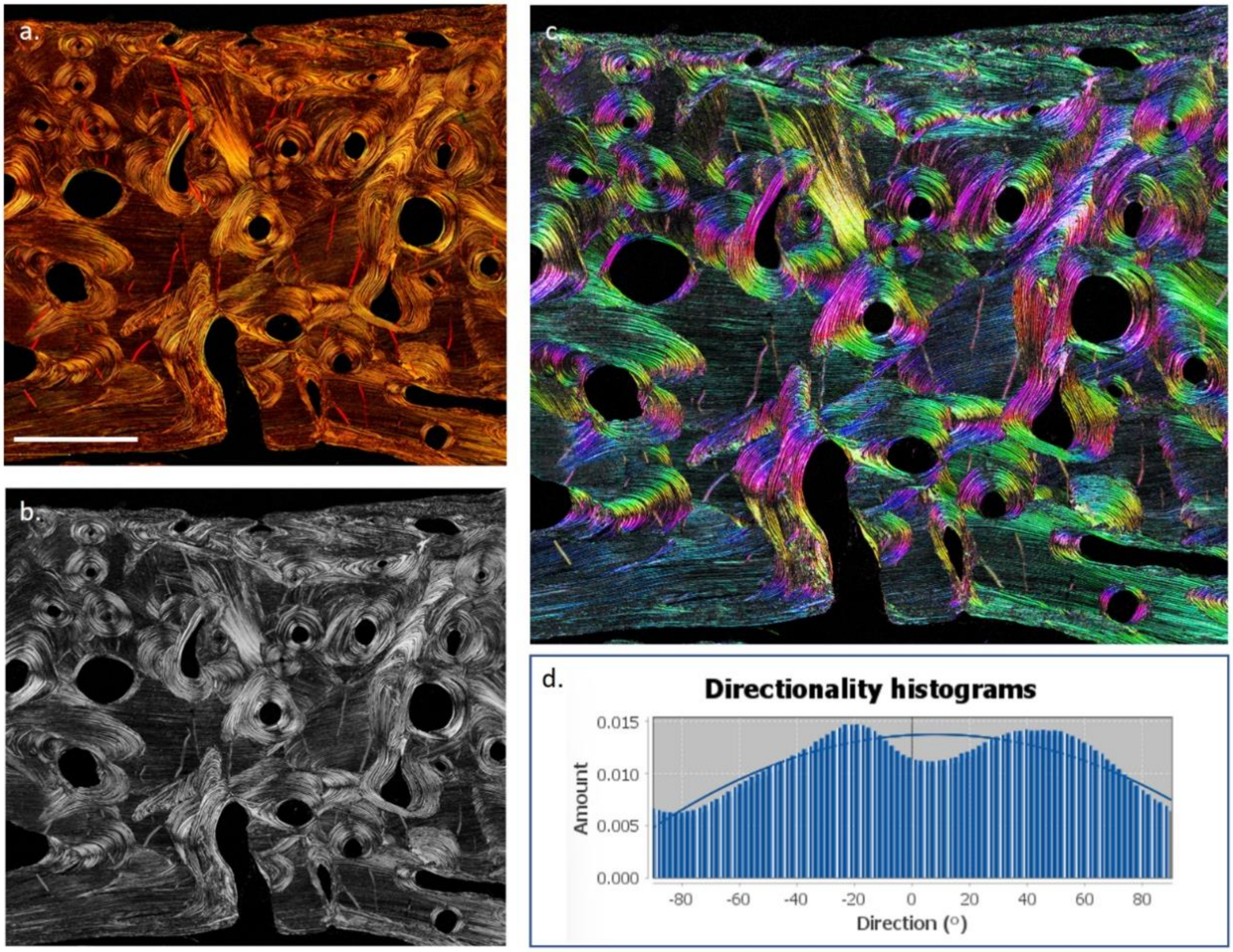

Supplementary Figure 2. Image processing steps for the assessment of fiber directionality and orientation distribution on a mandibular sample. Scale bar: 500µm.
